# Integrating parent report, observed behavior, and physiological measures to identify biomarkers of sensory over-responsivity in autism

**DOI:** 10.1186/s11689-025-09597-6

**Published:** 2025-03-17

**Authors:** Apurva Chaturvedi, Sapna Ramappa, Ariana Anderson, Megan Banchik, Urvi Shah, Michelle Craske, Shulamite Green

**Affiliations:** 1 Department of Psychiatry & Biobehavioral Sciences, University of California, Los Angeles, USA; 2 UCSF School of Medicine, University of California, San Francisco, USA; 3 Department of Psychology, University of California, Los Angeles, USA; 4 Ahmanson-Lovelace Brain Mapping Center, Semel Institute of Neuroscience and Human Behavior, University of California, 660 Charles E. Young Drive South, Los Angeles, CA 90095 USA

**Keywords:** Sensory processing, Autism spectrum disorder, Physiology, Heart rate, Masking, Behavioral inhibition, Sensory over-responsivity

## Abstract

**Background:**

Sensory over-responsivity (SOR) is a heightened reaction to environmental stimuli commonly seen in autism spectrum disorder (ASD) which impacts daily functioning. Parent-reported and observed behavioral assessments are used to study SOR, but show limited associations with each other, possibly because they measure different aspects of SOR or because children inhibit their responses during standardized assessments. Physiological measures provide an objective measure of sensory reactivity, and atypical heart rate (HR) responses to aversive stimuli have been shown to be related to SOR in ASD youth. This study aimed to compare how reported and observed measures of SOR predict HR and to examine if the level of reported behavioral inhibition in ASD youth affects how observed SOR behaviors correlate with physiological reactivity.

**Methods:**

Participants were 54 typically developing (TD) and 83 ASD youth, ages 8–17, who completed a standardized behavioral assessment of SOR while electrocardiogram recordings were collected. Participants’ parents also reported on their child’s SOR symptoms and behavioral inhibition.

**Results:**

ASD youth showed lower inter-beat-intervals (IBI; higher HR) across all auditory and tactile stimuli. For ASD youth, parent-reported SOR interacted with observed SOR to predict HR changes across the stimulation periods, indicating that ASD participants whose parents reported they had high SOR in their daily life, *and* showed high observed SOR in the lab assessment, exhibited reduced HR deceleration (orienting) after the onset of the stimulus and subsequent increased HR acceleration. Finally, we found that ASD participants who had lower parent-reported behavioral inhibition had a stronger correlation between observed SOR behavior and atypical HR responses.

**Conclusions:**

Results support prior findings that increased HR responses to aversive stimuli is related to both ASD and SOR. Furthermore, observed and parent-reported SOR interacted to predict HR, suggesting that a multi-method approach may best capture the extent of SOR for an individual. However, observed SOR measures may be most accurate for ASD youth who are less likely to inhibit their behavioral responses. This study illustrates the importance of integrating multiple measures of sensory reactivity to identify SOR. HR measures of sensory reactivity have the potential to serve as a biomarker of SOR across a diverse range of individuals.

## Background

Individuals diagnosed with autism spectrum disorder (ASD) experience significant sensory processing challenges [18, 45]. In particular, sensory-over responsivity (SOR) which entails an exaggerated negative response to aversive environmental stimuli, such as loud sounds, bright lights, and different textures [18, 25], can make it difficult to engage in daily activities and social interactions and is a major impediment to quality of life [18, 21, 45]. SOR is highly prevalent in ASD, affecting at least 50–70% of individuals diagnosed with ASD [6, 44].

Despite increasing interest in SOR since sensory processing differences were added to the DSM-V criteria for ASD in 2013, major challenges still exist in accurately measuring SOR [33]. Historically, SOR in children has been most commonly measured through parent report questionnaires [10, 14] such as the Sensory Profile [28], the Sensory Processing Measure [24], and the Sensory Processing 3-Dimensional Inventory [29, 39]. Parent report measures can give insight into a child’s responses to commonly encountered stimuli and environments, which can go beyond what is observed in a lab setting, thereby offering a broader perspective of a child’s SOR experience in a quick, more accessible, and less labor-intensive manner compared to lab assessments (e.g., [11, 22, 35]). However, parent reports can be influenced by parents’ expectations about their child, their knowledge of a “normative” comparison group, and their child’s developmental history or other symptoms [42, 45].

In contrast, observed lab assessments could potentially offer a standardized, more objective measure of SOR. Examples include the Sensory Processing Assessment (SPA) and Sensory Processing 3-Dimensional Assessment (SP3-D), which are designed to code responses to standardized sensory stimulus presentation in young children through adolescents [2, 31]. Objective, standardized observations in a controlled setting could improve cross-comparison across individuals. However, the standardized controlled nature of these lab assessments may also not mimic dynamic, real-world environments, therefore not giving insight to a holistic view of an individual’s SOR and the degree their SOR impacts them daily [35].

Interestingly, previous literature has shown that measures of SOR across lab assessments and parent reports are usually not correlated [35, 39, 42]. This disconnect could be due to the two measures capturing different aspects of SOR: the parent-reported measures may capture more “trait”-based, ecologically valid behavior across different environments whereas the lab assessment might identify more of a “state”-based response to particular stimuli in a standardized environment [35]. Relatedly, some participants may inhibit or “mask” their responses in a standardized setting so that even if they are affected physiologically or emotionally by stimuli, they may not react behaviorally. This idea is supported by data showing that SOR behaviors on a lab assessment are negatively correlated with age, whereas parent report is not– suggesting that children may improve their ability to inhibit behavioral responses as they get older, but parents, who see them in a wider range of natural environments, may still pick up on their aversive reactions to certain sensory stimuli [34, 41]. Decreases in behavioral responses with age could also indicate an increased ability to mask sensory responses as children get older. Masking is common in autistic individuals to meet social norms, avoid stigmas associated with autism, and suppress behaviors seen as atypical, including SOR responses [30, 34].

This complex intersection between environmental inputs, internal affective experience, biological reactivity, and behavioral output indicates that multi-dimensional methods are needed to better identify and measure SOR across diverse individuals with different internal and external responses that can vary based on their age, temperament, regulatory skills, etc. [11, 22, 35]. Physiological measures of arousal show promise as an objective, quantifiable measure of sensory reactivity which can potentially be used to identify SOR, as previous literature has found that ASD youth have physiological differences in heart rate (HR) compared to their TD counterparts in response to aversive stimuli [23, 13, 47]. Elevated HR responses to aversive sensory stimulation also appear to be specifically related to SOR in autism [23] and HR responses predict SOR symptoms over and above other general symptoms of arousal, such as anxiety [13]. HR is an objective measure of arousal that is non-invasive, easy to collect across ages and symptom severity levels [15], and could help identify children who are inhibiting their behavioral responses but who still over-react to stimuli at a biological and affective level. More specifically, the time between heartbeats, also known as the inter-beat-interval (IBI), can be used to measure second-by-second changes in HR responses. A recent study by Jung et al. [23] using IBI found that typically developing youth and ASD youth with low SOR show a typical deceleration in HR in response to an aversive sensory stimulus, known as an “orienting” response that allows initial processing of the basic properties of a stimulus [8]. In contrast, ASD youth who had high SOR showed reduced orienting responses followed by increased acceleration, suggesting that lack of initial processing might lead to transitions into fight-or-flight mode more often and more quickly [23]. With this literature in mind, combining physiological measures with observed behavior may inform understanding of which ASD youth dislike aversive stimuli, but do not show it in their outward behavior due to active inhibition.

The first goal of this study was to replicate prior findings in a larger sample showing that physiological measures, like IBI, can be used to differentiate between ASD and TD youth, and relate to SOR; we hypothesized that ASD youth would have lower IBI responses (faster HR) than TD youth and that within ASD, SOR would predict more atypical IBI responses, as in prior research. We further aimed to examine how reported versus observed SOR behavior uniquely predict an objective, physiological reactivity measure of SOR in ASD youth. We expected that observed and parent-reported SOR would both predict unique variance in physiological reactivity given the historical lack of correlation between these two types of measures. Finally, we aimed to determine if a child’s ability to inhibit their behavior affects the extent to which their HR correlates with observed behaviors in the lab, thereby providing insight into why lab assessment and parent reported measures do not correlate. We hypothesized that children with more behavioral inhibition would be more likely to mask their dislike of sensory stimuli, such that heart rate may not correlate with the behaviors that are observed in the lab (i.e., these children would show low observed behavior regardless of their HR responses and true SOR experiences). However, for children with less behavioral inhibition, heart rate would correlate more highly with observed behavior as youth with higher heart rate who experience the stimuli as more unpleasant would be more likely to demonstrate aversive responses in their outward behavior.

## Methods

### Participants

Participants included 54 TD (35 male) and 83 ASD (58 male) youth between the ages of 8 through 17 years old (M = 12.40, SD = 2.72). All participants had an intelligence quotient (IQ) of 70 or above. There were no significant group differences in sex, race/ethnicity, age, baseline HR, or body mass index (BMI; see Tables 1 and 2). The TD group did have a significantly higher mean IQ than the ASD group (see Table 2), so Full-Scale Intelligence Quotient (FSIQ) was tested as a covariate in all diagnostic group comparisons and included in the final models when significant at *p* <.10. Thirty of the ASD participants were using psychotropic medications, including stimulant medication (*n* = 25), selective serotonin-reuptake inhibitors (SSRIs; *n* = 17), anti-epileptics (*n* = 1, Trileptal), and mood stabilizers (*n* = 2). The TD group had no participants on medication. Medication status was tested as a covariate in all analyses and had no effect on any of the results. The study was approved by the UCLA Institutional Review Board, and informed consent and assent were obtained from the participants and their parents.

**Table 1:** Group differences in sex, race, and ethnicity

	ASD		TD			
Sex Assigned at Birth	n	%	n	%	^χ2^	*p*-value
Female	25	30%	19	35%	0.33	0.57
Male	58	70%	35	65%		
Total	83		54			

Race and Ethnicity	n	%	n	%	^χ2^	*p*-value
White, Hispanic or Latino/a	21	25%	8	15%	9.69	0.21
White, not Hispanic or Latino/a	29	35%	17	31%		
Black or African American, not Hispanic or Latino/a	5	6%	4	7%		
Multiracial	20	24%	13	24%		
Asian	6	7%	10	19%		
Unknown race and unknown ethnicity	2	2%	2	4%		

**Table 2:** Group differences in demographic data and study measures

	ASD		TD		Mean Differences	
Measure	Mean	SD	Mean	SD	t	*p*-value
Age	12.61	2.77	12.07	2.65	1.16	0.25
BMI	21.04	5.18	19.71	4.62	1.17	0.13
FSIQ	106.24	16.53	112.85	11.85	-2.54	0.01
Baseline HR	84.62	15.60	81.94	10.53	.11	0.27
Parent Reported SP3D Total Score	13.0	11.43	1.87	3.23	6.96	< 0.001
Parent Reported SP3D Tactile Score	6.30	4.84	1.04	1.66	7.61	< 0.001
Parent Reported SP3D Auditory Score	5.69	5.96	0.66	1.64	5.99	< 0.001
Observed SP3D Total Score	1.60	1.59	0.50	0.91	4.62	< 0.001
Observed SP3D Tactile Score	1.01	1.15	0.30	0.69	4.11	< 0.001
Observed SP3D Auditory Score	0.37	0.73	0.15	0.36	2.11	0.04
Observed SP3D Auditory and Tactile Score Combined	1.36	1.40	0.44	0.82	4.46	< 0.001
CBCL Emotion Dysregulation Index (behavioral inhibition)	8.98	6.57	1.81	2.4	7.67	< 0.001
Parent Reported SCARED Total	21.94	15.13	6.81	7.00	6.96	< 0.001

### Observed behavioral measures

Observed sensory over-responsivity was evaluated using the Sensory Processing 3-Dimensional (SP3-D) Assessment, which measures behavioral responses to ecologically relevant sensory stimuli such as bright lights, loud sounds, and rough textures [31]. Experimenter-administered tactile and auditory modulation tasks were used for the purposes of this study. While the original SP3-D includes only child-administered tactile and auditory stimuli, for this study the same stimuli were first administered by the experimenter for 30-seconds each to allow for standardized coding of simultaneous behavioral and physiological responses [35]. For the tactile tasks, participants were exposed to experimenter-administered brushing of the arm for 30 s each with a paintbrush and the bumpy plastic handle of a pedicure scrubber. They also experienced 30 s of a sponge toothette rubbed around their lips. Timing of each stimulus was standardized at one stroke per second from wrist to elbow for the arm stimuli, and one second per rotation around the lips for the toothette. Finally, participants were asked to take a toy out of slime. This final task was coded only for behavior, to increase the variability of tactile SOR scores, but was not included in psychophysiological analyses as individual variability in motion while interacting with the slime interfered with collecting standardized HR measurements. For the auditory tasks, participants were exposed to a video of an experimenter playing cymbals, a cymbal and a stick, and a whistle for 30 s each. In between each stimulus, participants experienced nine seconds of rest, while they sat still and looked at a fixation cross.

To standardize the scoring, all scorers were trained according to the SP3-D administration manual by a licensed psychologist, who also acted as the master coder, based on the SP3-D administration manual (unpublished; see [31]), as well as consultation with one of the developers of the assessment. SP3-D sensory behaviors were scored using standardized scoring guidelines in consensus with a master coder and with developers of the assessment. Inter-rater reliability was established by reviewing scores with the master coder, where each scorer had to reach 90% inter-rater reliability prior to scoring on their own, and then at least 20% of assessments were co-scored with the master coder. The inter-rater reliability for these assessments was 96%. According to the assessment scoring created by Tavassoli et al. [42], atypical aversive responses were recorded via a binary code. Observed SOR behaviors during a task (e.g., clenching fists or grimacing when touched by a tactile brush or covering ears in response to the auditory sounds) were coded as 1. If no SOR behaviors were observed, then the task was scored a 0. Possible scores ranged from 0–4 for tactile, 0–3 for auditory, and 0–7 for the total score.

### Reported measures

On the same day as the sensory assessment, participants’ parents completed the Parent Sensory Processing 3-Dimensions Inventory. In this survey, parents indicate the total number of daily environmental stimuli that elicit SOR responses across visual, tactile, and auditory domains. For this study, only SOR behaviors for the tactile and auditory domains were calculated, with possible scores ranging from 0 to 44 [39].

Parents completed the Child Behavior Checklist (CBCL) about their child [1]. Parents responded, “not true,” “somewhat or sometimes true,” and “very true or often true” for questions relevant to their child’s behaviors and emotions, such as anxiety, depression, or social problems. From this questionnaire, we used the CBCL Emotion Dysregulation Index (EDI; [36]) to measure behavioral inhibition. The EDI is calculated from 18 questions relevant to behavioral inhibition and emotion dysregulation, such as indicating whether their child “gets in many fights”, “destroys his/her own things”, “screams a lot”, has “temper tantrums”, etc.

The CBCL EDI has possible scores ranging from 0 to 36. There is a negative relationship between the EDI score and level of behavioral inhibition, where higher scores indicate less inhibition. Additionally, participants’ parents completed the Screen for Child Anxiety Related Emotional Disorders (SCARED), a total anxiety parent-reported questionnaire that has a list of 41 questions related to general anxiety disorder, panic disorder or significant somatic symptoms, separation anxiety disorder, social anxiety disorder, and school avoidance [7]. A score of 25 and above indicates the presence of an anxiety disorder.

### Physiological measures

During the SP3-D assessment, HR for each task was measured via electrocardiogram (ECG) while participants were seated. Participants had two electrodes connected to the BIOPAC MP150 system, allowing physiological data acquisition at a sampling rate of 2000 Hz. One electrode was placed on the right side of their body below the collarbone, and the second electrode on the left side of their body just below their rib cage. HR values and the IBI in milliseconds were extracted via the program Autonomic Nervous System Laboratory (ANSLAB) to be compared across groups. ANSLAB utilizes an algorithm which detects the R peaks of every heartbeat for the entire data file. The time periods of each of the SP3-D tasks were manually indicated by the experimenter. After the ANSLAB identified the R peaks for each stimulus period, the experimenters quality-checked the data to verify the correct location of the R peaks. Any non-usable data, such as data that was not acquired due to electrode failure or data where R peaks could not be identified due to motion artifacts, was not included in the final analysis. If a time period had up to three missing R-peaks, ANSLAB could still apply its algorithm to calculate IBI. However, if more than three R peaks were missing, then the program could not calculate IBI, in which case that participant was excluded from relevant analyses (6 participants in tactile and 5 in auditory as noted in Psychophysiological Analysis section of the Methods). Once the correct R peaks are identified, ANSLAB calculates the IBI as the time between heartbeats for a given time period, in milliseconds.

Baseline HR was measured for three minutes at the beginning of the assessment. IBI for each task was calculated for 10 time intervals after stimulus onset in the following manner: 0–1 s, 1–2 s, 2–3 s, 3–4 s, 4–5 s, 5–10 s, 10–15 s, 15–20 s, 20–25 s, 25–30 s. This method of dividing up the stimulus period is consistent with previous literature that has examined ECG responses to sensory stimuli [16, 23, 27, 46]. Change in IBI for each interval was calculated as the IBI during each of the time intervals minus the mean IBI 5 s before stimulus onset, during the fixation rest period. Higher IBI values indicate an increased time between heartbeats, which equates to a *slower* HR, whereas lower IBI values indicate a *faster* HR. The orientation phase was considered to be 0–4 s during which time HR was decelerating on average, and the acceleration phase was 4–10 s, with the remaining time (10–30 s) considered a habituation phase.

### Psychophysiological analysis

IBM SPSS Statistics version 29 software was used to run the statistical analyses. To analyze diagnostic group differences in IBI responses, a repeated-measures ANOVA was conducted with diagnostic group (ASD or TD) as a between-subjects factor and stimulus type (the three tasks for each sensory domain) and time (each of the 10 intervals across the 30-second task, as described in the Methods section) as within-subjects factors. Additionally, FSIQ, age, sex, CBCL EDI, SCARED scores, and BMI were tested as covariates and kept in the analysis wherever significant at *p* <.10.

To analyze the effect of reported and observed measures of SOR on IBI responses, similar repeated-measures ANOVAs were conducted within the ASD group only with stimulus type and time as within-subjects factors. Here, in addition, for the tactile analysis, total parent-reported tactile SOR and total observed tactile SOR as well as the interaction between the two were entered as between-subjects factors. Similarly, in the auditory analysis, total parent-reported auditory SOR and total observed auditory SOR as well as the interaction between the two were entered as between-subjects factors. As in the previous analyses, FSIQ, age, sex, and BMI were tested as covariates and kept in the analyses wherever significant at *p* <.10. We chose to conduct these analyses in the ASD group only since previous literature has shown that there is less SOR variability within the TD group, and/or SOR-related results are driven by outliers, similar to our TD sample [6, 13, 20, 35].

To analyze the extent of reported inhibition on the relationship between observed SOR behaviors and IBI responses, a linear regression analysis was conducted within the ASD group to predict total (tactile plus auditory) observed SOR scores from the interaction of (1) the orientation slope with behavioral inhibition and (2) the acceleration slope with behavioral inhibition. The slope from 0– 4 s post-stimulus onset was calculated for the orientation phase, and 4–10 s post-stimulus onset for the acceleration phase. A negative slope in the orientation phase indicates less orientation (i.e., less of a typical heartbeat-slowing response at the onset of a stimulus), and a positive slope indicates more orientation or more slowing. A negative slope in the acceleration phase indicates *increased* HR acceleration, and a positive slope indicates HR *slowing* instead of acceleration. Mean-centered main effects (orientation slope, acceleration slope, EDI score) and covariates that showed a significant correlation with orientation and acceleration slopes (BMI, age, FSIQ, and parent-reported SOR) were entered in a first step, and the interaction terms were entered in a second step, in a regression equation predicting total observed SOR scores (see Table 3).

**Table 3:** Regression table for the Interaction between Regulation and HR on observed SOR behaviors

	Standardized B Final Model	Std. Error	F Change	ΔR2
**Step 1**:			.39	.09
Age	.09	.06		
BMI	−.08	.04		
Orienting Slope	−.001	9.45		
Acceleration Slope	−.07	3.11		
Emotion Dysregulation Index (EDI)	.18	.03		
Parent Reported Auditory/Tactile SOR	−.20	.02		
**Step 2**:			4.83*	.12*
EDI* Orienting Slope	−.27*	1.56		
EDI* Acceleration Slope	−.25*	.47		

* *p* <.05, B final model indicates the standardized beta-values for each variable included in the final model.

Three ASD participants were removed from the tactile domain analyses and one from the auditory analyses due to being extreme outliers (more than 3 times above or below the inter-quartile range). Additionally, three TD participants were missing ECG data for the tactile tasks, and two TD participants were missing ECG data for the auditory tasks. Three ASD participants were missing ECG data for both the tactile tasks and auditory tasks. Thus, there were 51 TD and 77 ASD participants in the final tactile analyses, and 52 TD and 79 ASD participants in the final auditory analyses.

## Results

### Baseline heart rate

An independent samples t-test showed that there were no significant diagnostic group differences in baseline HR (t(135) = 1.11, *p* =.27). In addition, baseline HR was not correlated with observed or reported SOR for the ASD group (r(81)=-0.001, *p* =.99, and r(76)=-0.11, *p* =.35, respectively). Baseline HR was negatively correlated with age for the TD group (r(52)=-0.35, *p* =.01), but no such correlation was found within the ASD group (r(81)=-0.08, *p* =.48).

### Diagnostic inter-beat-interval differences (heart rate acceleration/deceleration)

Repeated-measures ANOVAs were conducted to compare diagnostic group differences in IBI responses after the onset of the tactile and auditory stimuli in the SP3-D assessment. For these analyses, the Mauchly’s Test of Sphericity was significant, suggesting that sphericity could not be assumed, so the Greenhouse-Geisser corrections were applied.

#### Group differences in responses to tactile stimuli

**Fig. 1:**
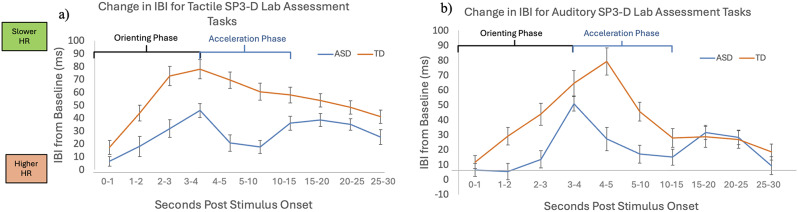
Diagnostic group differences in heart rate responses. Figures depict differences in the autism spectrum disorder (ASD) group and typically developing (TD) group for inter-beat interval (IBI) change up to 30 s post-stimulus onset compared to the 5-seconds of inter-trial interval (ITI) rest prior to the stimulus, averaged across all three (**a**) tactile stimuli and (**b**) auditory stimuli during a standardized observed behavioral assessment

There was a significant main effect of time (F(5.14, 116) = 3.14, *p* =.01), showing that HR changed significantly across the period of tactile stimulation. There was also a significant main effect of diagnostic group across the tactile SP3D trials (F(1,124) = 17.78, *p* <.001), and there was an overall time by diagnosis interaction (F(5.1,116) = 4.28, *p* <.001). These results indicated that the ASD group had faster HR (less time between heartbeats) across the tactile stimuli, and that HR slopes remained flatter over time for the ASD group (see Fig. 1a). Additionally, there was a significant main effect of sex (F(1,124) = 7.01, *p* =.01) where females had a faster HR across all tactile stimuli, and a significant time by age interaction (F(5.1,116) = 2.70, *p* =.02), where younger participants sped up HR faster in the second half of the task. There were no other significant main effects or interactions with time.

A post-hoc least significant difference (LSD) analysis of simple effects indicated that the ASD group had significantly faster HR than the TD group during the orientation phase: 1–2 s (*p* =.03), 2–3 s (*p* <.001), 3–4 s (*p* <.001) and the acceleration phase: 4–5 s (p = <.001), 5–10 (*p* <.001). There were also significant differences in in the habituation phase: 10–15 s (*p* =.01), 15–20 s (*p* =.03), 20–25 s (*p* =.03), and 25–30 s (*p* =.04). Taken together, results indicated that the ASD group had reduced slowing during the orientation phase of 0–4 s but faster increases in HR in the acceleration phase of 4–10 s. Although habituation occurred in the ASD group, HR still remained higher than the TD group across the habituation period (see Fig. 1).

#### Group differences in responses to auditory stimuli

Similarly to the tactile analysis, there was a significant main effect of time across the auditory stimuli (F(4.77, 120) = 5.72, *p* <.001), a significant main effect of diagnostic group on HR responses to auditory stimulation (F(1,128) = 5.67, *p* =.02), and a time by diagnosis interaction (F(4.77,120) = 6.77, *p* <.001). As in the tactile analysis, the ASD group had a higher HR (less time between heartbeats) compared to the TD group (see Fig. 1b) and a flatter overall slope. There was also a significant main effect of age (F(1,128) = 7.35, *p* =.01) and a time by age interaction (F(4.7,120) = 2.32, *p* =.045), where younger participants had overall higher HR responses as well as differences in change over time, best explained by a stronger orienting (deceleration) response but and then faster acceleration throughout the rest of the stimuli. There were no other significant main effects or interactions with time.

The post-hoc LSD simple effects analysis showed a significantly lower change in IBI, indicating higher HR responses, for the ASD group than for the TD group during the orientation phase: from 1 to 2 s (*p* =.009), 2–3 s (*p* =.003), and during the acceleration phase: from 4 to 5 s (p = <.001), and 5–10 s (*p* =.003) after stimulus onset compared to stimulus baseline. Thus, similarly to the tactile analysis, higher HR in the ASD group appeared to be driven by slower orientation and faster acceleration after stimulus onset.

### The relationship of reported and observed SOR on IBI responses

Repeated-measures ANOVAs were conducted within the ASD group to determine the relative contributions of observed and reported measures of SOR on HR responses to tactile and auditory stimuli. The Mauchly’s Test of Sphericity was significant, so for these analyses, the Greenhouse-Geisser corrections were applied.

#### Relationship between SOR and IBI responses to tactile stimuli

For the tactile stimuli, there was a significant two-way interaction between observed SOR responses and time (F(4.96,64) = 3.27, *p* =.01), indicating that participants with higher observed tactile SOR had reduced orientation and faster acceleration compared to those with lower observed SOR. There was also a trend towards a three-way interaction effect between parent-reported tactile SOR, observed tactile SOR, and time (F(4.96, 64) = 1.96, *p* =.08). There was a significant time by age interaction (F(4.96,64) = 2.83, *p* =.02), where younger participants increased their HR more in the second half of the stimuli and habituated less compared to older participants. There were no other significant main effects or interactions with time.

**Fig. 2:**
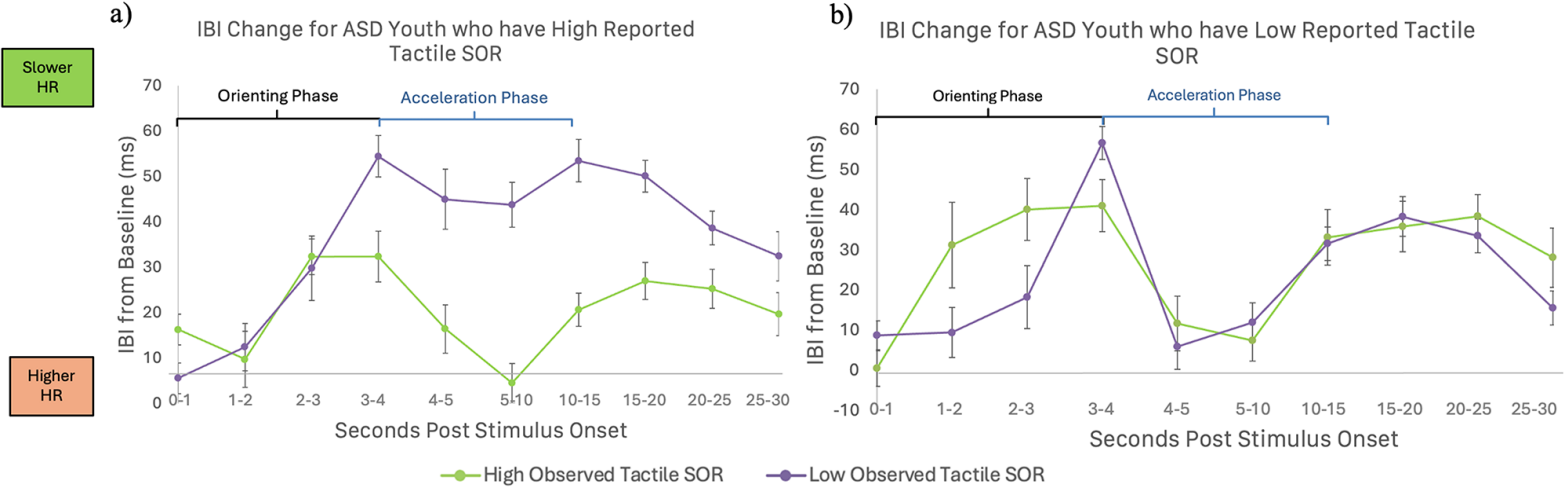
Interaction between parent-reported and observed sensory over-responsivity (SOR) predicting heart rate responses to tactile stimuli. Figures depict inter-beat interval (IBI) change up to 30 s post-stimulus onset compared to the 5-seconds of inter-trial interval (ITI) rest prior to the stimulus, for (**a**) autistic (ASD) participants whose parents reported them having high tactile SOR and (**b**) ASD participants reported to have low tactile SOR, divided with a median split. Each figure shows differences between youth who showed high observed tactile SOR (green) or low observed tactile SOR (purple) during the SP3-D assessment, divided with a median split. In the group who had high parent-reported SOR only, youth with high observed tactile SOR showed reduced heart rate deceleration and increased acceleration in response to sensory stimulation compared to those who had low observed SOR

To interpret the three-way interaction, a second repeated-measures ANOVA was conducted as a follow-up, to test the two-way interaction between time and observed SOR separately for high and low parent-reported SOR groups (determined by a median split). This analysis indicated that there was a significant observed SOR by time interaction only for participants with higher parent-reported tactile SOR (Fig. 2a; F(4.63,25) = 2.50, *p* =.04). Conversely, for participants with lower parent-reported tactile SOR, observed tactile SOR was not related to IBI change over time (Fig. 2b; F(4.52,30) = 1.50, *p* =.20). Thus, participants with the most atypical orienting and acceleration responses were both rated by their parents as having high tactile SOR and showed more tactile SOR behaviors during the SP3D assessment.

#### Relationship between SOR and IBI responses to auditory stimuli

**Fig. 3:**
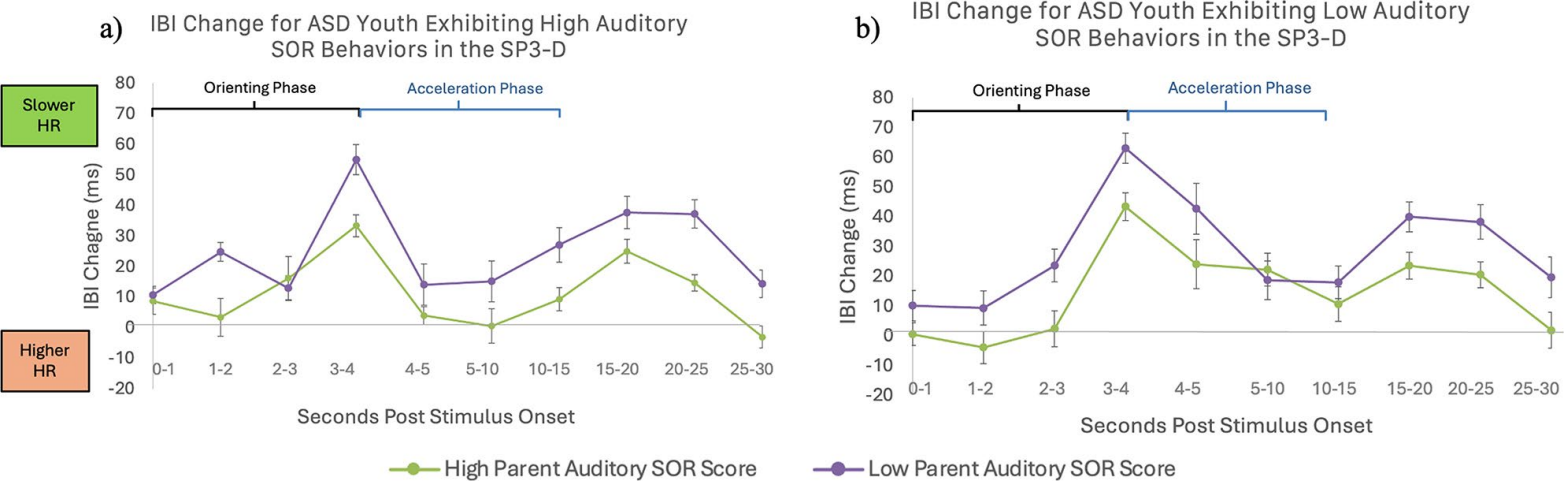
Interaction between parent-reported and observed sensory over-responsivity (SOR) predicting heart rate responses to auditory stimuli. Figures depict inter-beat interval (IBI) change up to 30 s post-stimulus onset compared to the 5-seconds of inter-trial interval (ITI) rest prior to the stimulus, for (**a**) autistic (ASD) participants had high observed auditory SOR and (**b**) ASD participants with low observed auditory SOR, divided with a median split. Each figure shows differences between youth who had high parent-reported auditory SOR (green) or low parent-reported auditory SOR (purple). In the group who had high observed SOR only, youth with high parent-reported auditory SOR showed reduced heart rate deceleration in response to sensory stimulation compared to those who had low parent-reported SOR

For the auditory domain tasks, there was a significant main effect of time (F(4.20, 66) = 2.64, *p* =.03, as well as a significant three-way interaction effect between parent-reported auditory SOR score, observed auditory SOR, and time (F(4.2,66) = 2.68, *p* =.03). To interpret the three-way interaction, as in the tactile analysis, a second repeated-measures ANOVA was conducted as a follow-up, to test the two-way interaction between time and observed SOR separately for high and low observed SOR groups (determined by a median split). This analysis indicated that for ASD participants with higher auditory observed SOR, higher parent-reported auditory SOR behaviors were more predictive of IBI changes across the stimulus (see Fig. 3). There was also a main effect of age (F(1,74) = 4.68, *p* =.03 showing that younger participants had higher HR responses across the stimuli compared to older participants. There were no other significant main effects or interactions with time. Thus, similar to in the tactile analysis, results indicated that participants who were both rated by their parents as having high auditory SOR, and showed more auditory SOR behaviors during the SP3D assessment, had the most atypically reduced orienting responses (Fig. 3).

#### Interaction between behavioral inhibition and HR on observed SOR behaviors

Given that the orientation and acceleration phases of HR response consistently showed both diagnostic group differences as well as relationships with SOR in this study and in our prior study [22], we calculated orientation and acceleration slopes to address our final aim, of understanding how children’s ability to inhibit or mask their aversive sensory responses may affect the extent to which HR predicts observed behavior. A linear regression analysis was conducted to determine the effect of behavioral inhibition on the relationship between observed SOR behaviors and average change in IBI. Age, BMI, and parent-reported SOR were included as covariates as they all showed a correlation with acceleration slope at *p* <.10 (*r* =.23, *p* =.047; *r* =.22, *p* =.06; *r*=-.28, *p* =.02, respectively).

**Fig. 4:**
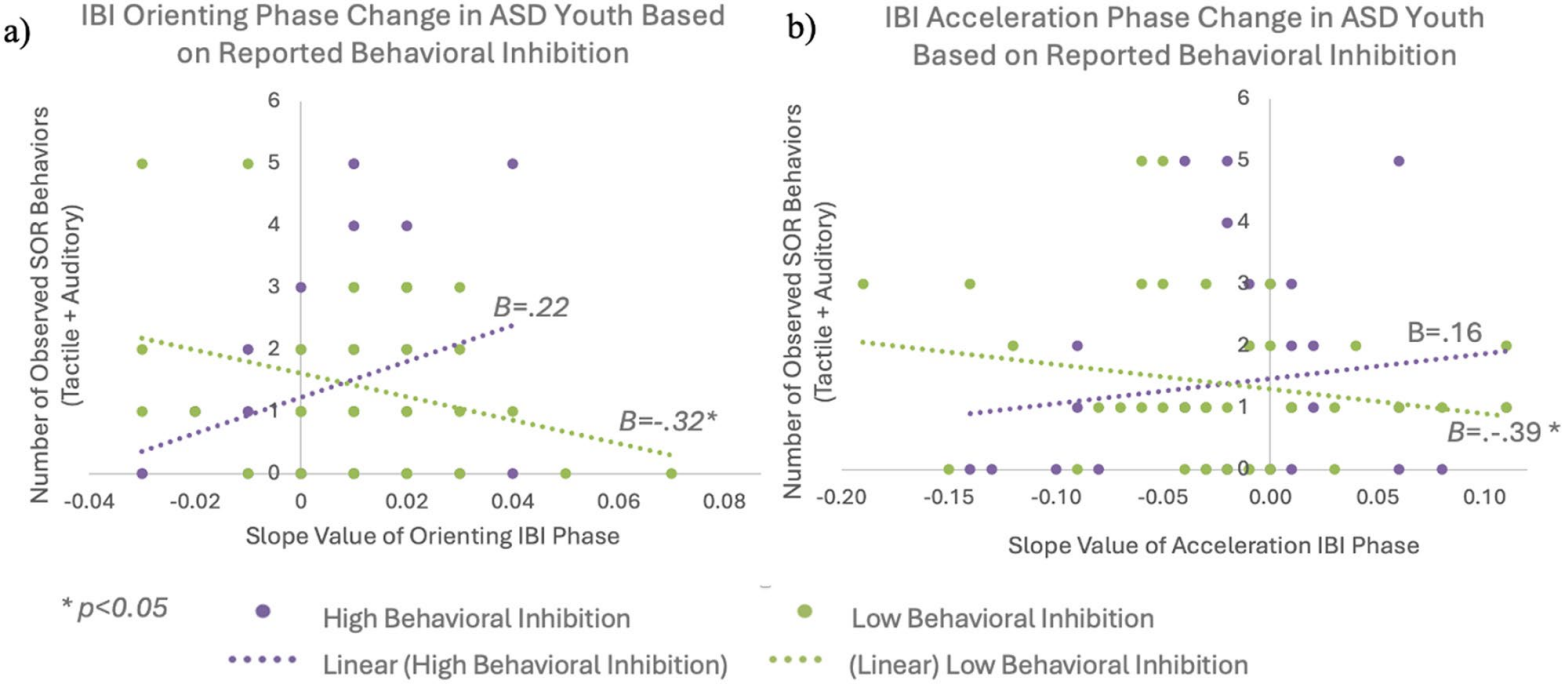
Interaction between heart rate responses and behavioral inhibition in predicting observed sensory over-responsivity. Figures demonstrate the relationship between (**a**) orienting slopes (i.e., rate of heart rate deceleration between 0–4 s after stimulus onset) and (**b**) acceleration slopes (i.e. rate of heart rate acceleration between 4–10 s after stimulus onset) with total auditory and tactile sensory over-responsivity (SOR) behaviors on a standardized observed assessment. In each scatterplot, the ASD participants are split into low (green) and high (purple) reported behavioral inhibition based on parent report. For both orienting and acceleration slopes, heart rate responses were more highly correlated with observed SOR for youth who were rated as less inhibited by their parents

A significant interaction effect was found between parent-reported behavioral inhibition (as measured by the CBCL EDI) and orienting slopes (i.e., speed of HR deceleration, *B = −.27*, *t(66)=* -2.31, *p* =.02) and acceleration slopes (*B = −.25*, *t(66)=* -2.30, *p* =.03) (F(8, 66) = 2.10, *p* =.01, R^2^ =.12) (See Fig. 4; Table 3).

Parent-reported behavioral inhibition was divided into high and low inhibition based on a median split of the CBCL EDI to interpret the interaction effect (see Fig. 4). Results indicated that as reported inhibition decreased (i.e., less inhibition), HR responses better predicted observed SOR behaviors in ASD youth (acceleration slope: *B*=-.39, *p* =.02; orienting slope: *B*=-.32, *p* =.04).

However, for those who were reported to be better inhibitors (parent-reported inhibition was higher), the relationship between HR and observed SOR was not significant (acceleration slope: *B* =.16, *p* =.37; orienting slope: *B* =.22, *p* =.24).

## Discussion

The goal of this study was to examine how diagnostic group as well as reported versus observed SOR behavior predict an objective, physiological measure of sensory reactivity. We further explored whether a child’s ability to inhibit or mask their aversive sensory responses affects the extent to which their HR correlates with observed behaviors in the lab, thereby providing insight into why lab assessment and parent-reported measures often do not correlate. Overall, findings replicated prior studies demonstrating that ASD youth show accelerated heart rates in response to aversive sensory stimuli compared to TD youth (e.g., [23, 47]). Furthermore, the results showed that parent reported and observed SOR behaviors actually interacted to predict HR responses to sensory stimuli in ASD. Finally, child behavioral inhibition skills affected the relationship between observed behavior and HR responses, suggesting important individual differences in the accuracy of SOR observed behavioral assessments.

To examine HR responses to aversive sensory information, this study focused on change in inter-beat interval from an inter-stimulus rest period to ten time periods post-stimulus onset. We found that the ASD group had overall higher HR responses than the TD group across all tactile and auditory stimuli, and that their HR responses changed over time differently across the stimulus periods. These results were mainly driven by differences during the first 10 s post-stimulus onset, known as the orienting and acceleration phases, with ASD youth showing reduced initial HR deceleration after the stimulus onset, followed by increased acceleration. Our results are consistent with prior literature which has shown that ASD youth, particularly those with high SOR, have faster HR responses to aversive sensory stimuli compared to TD youth, and that these higher responses are driven by reduced HR deceleration in the orienting phase and then increased acceleration post-stimulus onset [23]. The orienting and acceleration phases after exposure to a stimulus are vital in understanding biological responses to the environment as potential defensive mechanisms [8]. The orienting phase is a slowing of HR that occurs in the first few seconds directly after exposure to a stimulus and is thought to allow time to process the basic properties of a stimulus and to detect a potential threat, allowing for preparation of a response if needed [8]. This is followed by an acceleration phase, in which HR speeds up as needed, for example, to respond to a threat as identified in the orienting phase. Finally, acceleration is typically followed by a habituation phase in which HR slows over time [8]. Consistent with prior research [23], the particular pattern of reduced orienting followed by increased acceleration in ASD versus TD youth suggests that ASD youth are responding in a more defensive manner, and possibly entering “fight or flight” mode due to a lack of initial, adaptive threat processing. These results align with other findings that have shown that reduced heart rate variability (HRV) relates to autonomic nervous system (ANS) differences in ASD and TD groups [26, 43]). Reduced HRV relates to the nervous system’s ability to modulate between “fight or flight” response and resting responses [26]. Longer or more intense orienting responses may similarly contribute to the TD group better modulating their responses to a changing environment compared to the ASD group.

Interestingly, in this study, there were no diagnostic group differences in baseline HR, only in response to aversive stimuli, suggesting that the increased physiological arousal was specific to sensory responses. This idea is supported by our results demonstrating that more atypical HR responses were related to higher scores on behavioral measures of SOR within the ASD group. Because prior studies have shown that observed and parent-reported measures are often not correlated [32, 35, 39, 42], we aimed not just to examine whether SOR generally related to HR responses, but to compare how observed versus parent-reported measures associated with this more objective, biological measure of sensory reactivity. Results showed an interaction effect such that both parent report and observed behavioral SOR measures predicted HR responses. Specifically, ASD youth who were reported as having high SOR in their day-to-day lives by their parents *and* exhibited a high number of observed SOR behaviors in the lab assessment, had the most atypical HR patterns in response to sensory stimuli. Notably, these SOR measures predicted HR responses to aversive sensory stimulation over and above other processes that are also associated with an increased HR, such as general ability to inhibit behavioral responses in autistic youth or anxiety, as we saw no significant interactions with the SCARED scores in this study’s analysis. Similar to the diagnostic group difference analysis, these SOR-related differences were mainly driven by the orienting and acceleration phases. ASD youth with both higher parent-rated and observed SOR had reduced orienting and increased acceleration responses to tactile stimuli, as well as reduced orienting responses to auditory stimuli. The interaction between parent-reported and observed behavior here is particularly notable given that the behaviors were observed simultaneous to the HR collection, so one would expect a stronger correlation between observed behavior and HR simply because they were measured in response to the same stimuli at the same time. Indeed, in the tactile condition, there was a main effect of observed behavior on HR slope, suggesting that observed behavior alone may be a better predictor of HR compared to parent report alone. However, upon follow-up analysis the relationship between observed behavior and HR was only significant for those youth with higher parent-reported SOR, which is consistent with the idea that both measures of SOR contribute important variance in predicting HR responses.

These results are generally consistent with previous literature that shows ASD youth who have higher parent-reported SOR have reduced HR orienting and increased acceleration compared to both ASD youth with lower SOR and TD youth [23, 47]. However, this study further extends these prior findings in a larger sample by integrating an observed measure of SOR which actually incorporates behavioral responses to the same sensory stimuli for which HR responses are measured. The fact that HR responses are best predicted by the interaction of observed and reported SOR compared to either alone suggests that these measures are complementary. For example, parent report may better capture a child’s “trait” SOR in terms of how reactive they are during everyday life which may be biased based on a parents’ comparison group or be out of date based on a child’s past behaviors [32], whereas the lab task may best provide a standardized measure that captures how reactive youth are to the particular stimuli presented in the lab, compared to other participants (e.g., [35]).

One notable potential drawback of measuring SOR through observed responses to a standardized lab task is that there are likely to be individual differences in the extent to which participants show their dislike of a stimulus through outward behavior. We aimed to test this hypothesis by examining whether the level of behavioral inhibition in ASD youth impacts how HR predicts observed behavior in the lab assessment. Our findings did indeed show that inhibition ability matters: results indicated that ASD youth who are reported as less inhibited by their parents have a stronger correlation between observed SOR behaviors and HR reactivity, with higher SOR predicting reduced orienting and increased acceleration. Observed SOR was less predictive of HR for ASD youth reported as being more inhibited. In other words, among those youth who have more behavioral inhibition, those with high SOR may have high HR responses *without* corresponding behavioral indicators of SOR. In fact, the effort of inhibiting behaviors could potentially drive HR even higher for these individuals, with the effort of masking their behavioral responses interacting with their high sensory reactivity in increasing HR responses. These findings suggest that observed behaviors (at least during standardized lab tasks) may be a more accurate measure of SOR in ASD youth who are less likely to inhibit their behavioral responses. For those who are more inhibited, a controlled lab setting may influence their ability to inhibit aversive responses, which aligns with previous research that has shown that context does impact sensory processing in ASD youth, where an individual’s likelihood of masking their sensory responses depends on the circumstances and level of sensory input in their environment, such as at home compared at school [9, 37]. Repressing natural behavioral responses depending on the context could also be indicative of masking to meet social norms depending on the context, a phenomenon that is common in some individuals with ASD [34]. For individuals who have a greater ability to inhibit behavioral responses, questionnaire data that reflects everyday functioning could therefore be better indicators of their internal SOR experience. Importantly, the results of this study also suggest that physiological markers may be a more objective measure of SOR that is less sensitive to individual differences in masking and behavioral inhibition and perhaps less affected by environmental context compared to behavioral measures.

There are many strengths of this study, including the relatively large sample size compared to prior studies examining physiological responses in autism [17, 23, 47]. Furthermore, we were able to integrate multiple measures of SOR, including behavioral, reported, and physiological, which allows for a more in-depth understanding of the sensory experience of ASD youth. Additionally, observed behavior and heart rate were acquired during the same lab tasks, allowing a unique opportunity to compare physiological and behavioral responses to the same stimuli. While this study notes some general limitations of standardized behavioral assessments, there were also some specific limitations of the SP3-D in measuring responses to auditory stimuli. Specifically, there was little variability in the observed auditory behavioral scores in this study compared to the observed behavioral tactile scores. The SP3-D assessment may detect more variability in tactile compared to auditory SOR simply because of the way the stimuli are administered: tactile stimuli are rubbed directly on the participants’ arms, which may be more salient and “close” compared to the auditory tasks of the assessment, which are played through a speaker and therefore can be experienced as more “distant.” This ability to consider aversive stimuli as close versus distant is a common emotion regulation strategy [40] and especially for individuals with higher inhibitory skills, could lead to more outward behavioral responses to tactile versus auditory stimuli. Potentially, simple changes such as presenting the auditory stimuli with headphones to increase the intensity could improve this difference in experience of closeness. Additionally, these differences in tactile versus auditory stimuli may also be why observed tactile responses had a larger overall effect on HR responses to tactile stimuli: one would expect that if observed behavior accurately captures variability in SOR, it should be more related to HR responses collected during the same stimuli compared to questionnaire responses that capture sensory responses in everyday life more generally.

An additional limitation is that this study does not consider sensory under-responsivity (SUR) or sensory seeking in relation to physiological measures. Although SOR was the focus here due to its key role in quality of life [18, 21, 45], SUR and seeking are also important in understanding a child’s entire sensory processing profile and extent of sensory reactivity [3, 5, 10, 35]. Future directions of this study could include analyzing physiological markers of these additional sensory processes to explore if heart rate changes are also indicative of sensory-seeking behaviors, where HR accelerates due to excitement and craving instead of alertness and defensiveness.

Finally, while we hope that the results of this study can provide insight into measurement of SOR with ASD individuals who have a wide range of verbal and cognitive abilities, all participants in the current sample had an IQ score above 70, therefore future research is necessary to determine whether a similar relationship exists between SOR and physiological reactivity in individuals with intellectual disability.

## Conclusion

The results of this study indicate that heart rate responses to sensory stimuli show promise as a quantitative measure of sensory reactivity that can both differentiate ASD from TD youth and detect individual differences in SOR within ASD youth. While parent-reported and observed behavioral measures both contribute important information to understanding SOR, they may be sensitive to context as well as to individual differences in behavioral inhibition. Combining reported, behavioral, and physiological measures of SOR provides important insight to understanding which ASD youth are showing their aversion to stimuli outwardly in their behavior and which may be inhibiting behavioral responses despite finding the stimuli very unpleasant. By incorporating all three measures to understand SOR in ASD, our study builds on previous literature that advocates for a multidisciplinary model of sensory processing [10, 11]. Notably, physiological markers offer the potential means to study SOR across varied individuals on the spectrum, including those with a wide range of ages, intellectual and verbal abilities, motor skills, and regulation. HR measures are also cheaper, less time-intensive, and put less burden on participants compared to other biological measures of SOR, such as neuroimaging studies [11], and may therefore be useful as treatment outcome measures. Accurate measurement of SOR has been an ongoing challenge in the field for many years [33], but is a major priority given the importance of identifying and treating sensory processing difficulties in autism. This study contributes to our understanding of the benefits and drawbacks of different methods of measuring SOR, emphasizes the importance of taking an integrated, multi-method approach, and highlights heart rate as a promising physiological measure that is sensitive to identifying sensory processing differences and could potentially be used across a diverse range of individuals.

## Data Availability

No datasets were generated or analysed during the current study.

## Abbreviations

SOR: Sensory over responsivity
ASD: Autism spectrum disorder
TD: Typically developing
SP3-D: Sensory Processing 3-Dimensional Assessment
CBCL: Child Behavioral Checklist
HR: Heart rate
IBI: Inter-beat-interval
ECG: Electrocardiogram
ITI: Inter-trial-interval
SSRIs: Selective serotonin-reuptake inhibitors
SCARED: Screen for Child Anxiety Related Emotional Disorders
EDI: Emotion Dysregulation Index
BMI: Body Mass Index
IQ: Intelligence Quotient
FSIQ: Full-Scale Intelligence Quotient
ANSLAB: Autonomic Nervous System Laboratory
ANOVA: Analysis of Variance
LSD: Least significant difference
HRV: Heart rate variability
ANS: Autonomic nervous system
SUR: Sensory under-responsivity
